# Clusters of *Lactobacillus* Strains from Vegetal Origins Are Associated with Beneficial Functions: Experimental Data and Statistical Interpretations

**DOI:** 10.3390/foods9080985

**Published:** 2020-07-24

**Authors:** Nacim Barache, Yanath Belguesmia, Rabia Ladjouzi, Farida Bendali, Djamel Drider

**Affiliations:** 1 Laboratoire de Microbiologie Appliquée, Faculté des Sciences de la Nature et de la Vie, Université de Bejaia, Bejaia 06000, Algeria; nacimbarache@gmail.com; 2 BIOECOAGRO Unit of Research N° 1158, Univ. Lille, INRAE, Univ. Liège, UPJV, YNCREA, Univ. Artois, Univ. Littoral Côte d’Opale, ICV-Institut Charles Viollette, F-59000 Lille, France; yanath.belgusmia@univ-lille.fr (Y.B.); Rabia.Ladjouzi@univ-lille.fr (R.L.)

**Keywords:** *Lactobacillus*, antagonism, lactic acid production, anti-adhesive properties, Caco-2 cells antioxidant activity, exopolysaccharides

## Abstract

Nine strains of *Lactiplantibacillus plantarum* and one strain of *Lacticaseibacillus paracasei* that were recently isolated from prickly pears, fresh figs and blackberries, which are traditionally and largely consumed fruits in Kabylia (north of Algeria), were studied here for their antagonism and antioxidant properties as well as for production of exopolysaccharides. With respect to their inhibitory properties, these strains were tested against three food representative pathogens including *Escherichia coli* ATCC 8739, *Staphylococcus aureus* 2S6 and *Listeria monocytogenes* 162. The antagonism of these pathogens was attributable to lactic acid production, present in the cell free supernatant, at concentrations ranging from 9 to 16.74 g/L. The anti-adhesive properties observed on polystyrene or eukaryotic Caco-2 cells were exerted in a strain dependent-manner. Indeed, the scores obtained ranged from 27% to 75% for *S. aureus* 2S6, 54% to 95% for *L. monocytogenes* 162, and 50% to 97% for *E. coli* ATCC 8739. The co-aggregation of these *Lactobacillus* strains with the aforementioned target bacteria appeared to be exerted in a strain-dependent manner, with noticeably the upmost rate for *Lb. paracasei* FB1 on *S. aureus* 2S6. Interestingly, these novel *Lactobacillus* strains were able to produce a large amount (315.55 to 483.22 mg/L) of exopolysaccharides, and showed a significant scavenging activity on the 2,2-di-phényl-2-picrylhydrazyle (DPPH) synthetic free radical with rates of 51% to 56%. Of note, the highest antioxidant activity was observed for *Lb. paracasei* FB1 using the culture supernatants, intact cells or the intracellular extract. The statistical analysis of these data using the principal component analysis (ACP) enabled us to establish three distinct clusters with potential applications as bioprotective and/or probiotic agents, following further evaluation.

## 1. Introduction

Foodborne pathogens represent a major health risk for consumers [1], causing a variety of foodborne diseases such as abdominal pain, diarrhea, fever, low blood pressure, vomiting and other gastrointestinal (GIT) symptoms [2,3]. Foodborne pathogens are considered as one of the most critical public health concerns spreading worldwide [4,5,6]. This unwanted spread of foodborne pathogens can negatively affect the whole economy. Indeed, according to the American Control Diseases Center (CDC) and FoodNet reports, bacterial pathogens such as *Listeria monocytogenes*, *Escherichia coli*, *Staphylococcus aureus* and *Salmonella*, associated with foodborne diseases, are responsible for huge economic casualties [7,8]. Antibiotics were then introduced and used for prophylaxis and treatment of bacterial GIT infections, with the aim to mitigate this imminent risk. However, their intensive use has enabled the emergence of human antibiotic resistant strains, which is a critical health challenge [9,10,11]. On the other hand, consumers are requesting more and more minimally processed foods with extended shelf-life, in which chemical additives are replaced by natural products endowed with safe and inhibitory activities [12,13]. Lactic acid bacteria (LAB) are natural and renewable sources offering various advantages including a hypocholestrolemic effect [14] and antioxidant activity [15], and can be used as preventive agents [16] or food bio-protective cultures [17]. Different studies pointed out the beneficial attributes of *Lactobacillus* strains, with emphasis on their capabilities to produce inhibitory molecules, or other molecules with benefits. With respect to food safety aspects, the *Lactobacillus* strains were steadily reported as natural means to inhibit foodborne pathogens, and alleviate oxidative damage in food system and human body, and further preventing related diseases [18,19,20,21].

Remarkably, probiotics, even at low concentrations, can inhibit the growth of intestinal pathogens through different mechanisms. They, indeed, can reduce their adhesion to intestinal epithelium, impact their capabilities to form biofilm, or impede their invasion process [22,23,24]. *Lactobacillus* can advantageously compete for resources available in the GIT [25]. For that scenario, LAB can deploy different strategies based on their abilities to produce organic acids, antimicrobial peptides such as bacteriocins, exopolysaccharides (EPS), or hydrogen peroxide [26,27]. Therefore, screening of new *Lactobacillus* strains endowed with such beneficial attributes is more than timely. Related to that, there is an increasing interest in unconventional sources such as traditional foods, which are steadily reported for their richness in *Lactobacillus* with probiotic and bio-preservative features [28,29,30].

The present and exhaustive study aimed at assessing and deciphering the antagonistic and antioxidant properties of *Lactobacillus* strains from prickly pears, fresh figs and blackberries, which are traditional fruits largely consumed in the north of Algeria.

## 2. Materials and Methods

### 2.1. Microorganisms

*Lactobacillus* strains used in this work were recently isolated from Algerian fruits [31]. They include *Lb. plantarum* M10 and M12 isolated from blackberries (*Rubus* sp.), *Lb. plantarum* F2, F3 and 2F8 isolated from fresh figs (*Ficus carica*); *Lb. plantarum* NCA3, NCA4, FB3, FB13 and *Lb. paracasei* FB1 isolated from prickly pears (*Opuntia ficus-indica*). Stocks of these strains were maintained at −20 °C in de Man Rogosa and Sharpe (MRS) broth (Conda, Madrid, Spain), containing 30% (*v*/*v*) of glycerol (Sigma-Aldrich, Schnelldorf, Germany). These strains were cultivated anaerobically (AnaeroGen™ 2.5 L, Anaerobic Gas Generator, Oxoid, Thermo, Hampshire, UK) for 18–24 h in MRS broth at 37 °C prior use.

The target strains were *E. coli* ATCC 8739 isolated from feces, *L. monocytogenes* 162 isolated from food [32], and the clinical isolate *S. aureus* 2S6, kindly provided by Khalil Amrane hospital (Bejaia, Algeria). These strains were aerobically grown at 37 °C in brain heart infusion (BHI) (Sigma-Aldrich) or in Luria–Bertani (LB) broth (Sigma-Aldrich) and stored at −80 °C.

### 2.2. Antibacterial Activity

Taking into account that strains used here were not bacteriocinogenic, we focused our study on their capabilities to produce lactic acid. Thus, inhibitory properties of these ten *Lactobacillus* strains were assessed against *E. coli* ATCC 8739, *S. aureus* 2S6 and *L. monocytogenes* 162 using both the well diffusion and the spot-on-lawn methods [33]. For the well method, 10 mL of BHI agar previously inoculated with the target strain at 10^6^ CFU/mL were added to the Petri plates. Once the medium became solid, wells were made and filled up with 50 µL of neutralized or non-neutralized cell free supernatants (CFS), gathered from *Lactobacillus* cultures. Notably, CFS neutralization was performed with 3M NaOH, adjusting its external pH to 6.5 (pH 6.5). For the spot-on-lawn test, 5 µL of *Lactobacillus* cultures were deposited on MRS plates, and incubated for 18 h at 37 °C. Afterwards, 10 mL of BHI agar (8 g agar/L) previously inoculated with the target strain, at 10^6^ CFU/mL, were added and incubated again for 18 h at 37 °C. After incubation, the plates were inspected, any inhibition zone around the wells or the spots was recorded, and diameters were measured [33].

### 2.3. Quantification of the Lactic Acid

The quantification of lactic acid produced by *Lactobacillus* strains was performed by high performance liquid chromatography (HPLC) by spectra system P1000XR (Thermo Fisher Scientific, Waltham, MA, USA) using a Fast Fruit Juice Column (50 mm × 7.8 mm, Phenomenex, Torrance, CA, USA). The mobile phase used was H_3_PO_4_ (0.05%, *w/w*), with a flow rate of 0.8 mL/min and a temperature of 55 °C. Supernatants from *Lactobacillus* cultures were collected after 8, 18 and 24 h of incubation at 37 °C in MRS broth, then centrifuged (8000× *g*, 10 min, 4 °C), and filtered through a filter of 0.2 µm/pore size. The volume of injected sample was 25 μL, a calibration curve of pure lactic acid (Sigma-Aldrich) with concentrations of 1, 5, 10, 15 and 20 g/L was carried out to establish the correlation between data obtained from chromatographic peaks area and produced lactic acid concentration. The peak corresponding to lactic acid, which is eluted at 5.32 min [34], was identified using the Azur software.

### 2.4. Biofilm Formation Assessment of Lactobacillus Strains on Polystyrene Tissue Culture Plates (TCP)

To assess the biofilm formation of the studied strains to polystyrene plate, a semi quantitative method was used, as previously described [35]. Briefly, 100 μL of each culture of *Lactobacillus* (10^8^ CFU/mL), grown in MRS broth, were added to the wells of sterile 96-well microplates already filled with 100 μL of tryptic soy broth (TSB) (Difco, Detroit, MI, USA). The microplates were left for 15 min under gentle stirring before being incubated at 37 °C. After 24 h, the cultures were aspirated and the non-adherent cells were removed by two washes of the wells with phosphate buffered saline (PBS, 10 mM, pH 7.2). Subsequently, 200 μL of 96% ethanol (Sigma-Aldrich, St Louis, MO, USA) were added to each well in order to fix the adherent cells. After 15 min of fixation, the wells were drained, dried and then stained with 0.1% (*w/v*) crystal violet (Biochem Chemopharma, QC, Canada) for 30 min. The stained cells were washed twice with 200 μL of PBS before extracting the dye with 200 μL of 96% ethanol. The number of cells was quantified using a microplate reader (ELX800, BioTek, Winooski, VT, USA) by measuring the absorbance (A) at 630 nm. According to the recommendations of Stepanović et al. [36], these strains were classified into four categories. Considering Ac as the absorbance of the control (sterile TSB), the following interpretations were applied; A ≤ Ac: non-adherent (non-biofilm producer), 2Ac ≥ A > Ac: weakly adherent (weak biofilm producer), 4Ac ≥ A > 2Ac: moderately adherent (moderate biofilm producer), and strongly adherent (strong biofilm producer): A > 4Ac.

### 2.5. Exopolysaccharide Production

The exopolysaccharide (EPS) production of *Lactobacillus* strains was evaluated according to the method described by van Geel-Schutten et al. [37]. Briefly, *Lactobacillus* strains were grown for 72 h at 37 °C in 30 mL of MRS broth supplemented with 2% (*w/v*) glucose. Bacterial cells were removed by centrifugation (6000× *g* for 20 min, 20 °C) and two volumes of 95% (*v*/*v*) cold ethanol (Sigma-Aldrich) were added to one volume of untreated CFS and maintained at 4 °C for 24 h to precipitate EPS. Then, the obtained precipitates were recovered by centrifugation (2000× *g*, 15 min, 4 °C), washed with distilled water and dried at 60 °C until constant weight was reached. The dried weight was then measured to determine the amount of EPS produced by the *Lactobacillus* strains [29].

### 2.6. Inhibition of Biofilm Formation by Lactobacillus CFS

Firstly, 50 μL of non-neutralized CFSs of *Lactobacillus* strains grown in MRS broth, and 50 μL of *E. coli* ATCC 8739, *S. aureus* 2S6 or *L. monocytogenes* 162 at 10^6^ CFU/mL, prepared as above-indicated were mixed and added to the wells of sterile 96-well microplates containing 100 μL of sterile TSB medium (Difco, Detroit, MI, USA). The microplates were left under gentle stirring for 15 min before their incubation for 24 h at 37 °C [35]. Of note, the tests were performed in triplicate and sterile MRS broth was used as a negative control. According to Shokri et al. [38], to evaluate the pathogen biofilm removal by CFSs of lactobacilli, the aforementioned target strains were grown at 37 °C for 24 h in the microplates wells, allowing them to form biofilms. Afterwards, 100 µL of non-neutralized CFSs from *Lactobacillus* strain were added to the wells and then incubated again for 4 h, at 37 °C. Each test was done in triplicate and sterile MRS broth (100 µL) was used as a negative control. Biofilm reduction was measured using the same steps as for the biofilm quantification previously reported [35].

### 2.7. Ultra-Structure Alterations as Visualized by Transmission Electron Microscopy (TEM)

Cultures of *E. coli* ATCC 8739, *S. aureus* 2S6 and *L. monocytogenes* 162 incubated at 37 °C for 18 h were centrifuged (8000× *g*, 10 min, 4 °C), and the resulting cells were suspended in non-neutralized CFS of *Lb. plantarum* 2F8 cultures, which presented antibacterial and anti-adhesive activities as well as upmost lactic acid production. Sterile MRS broth (pH 6.5) was used as a control. All suspensions were incubated for an additional 18 h at 37 °C. Samples were collected from each suspension and the cells were recovered by centrifugation (8000× *g*, 10 min, 4 °C) as a small pellet. For transmission electron microscopy (TEM), the pelleted cells were fixed with 2.5% (*v*/*v*) glutaraldehyde solution and 0.1 M (*v*/*v*) of cacodylate buffer (pH 7.4) and prepared on a Formvar film of 300 square mesh, nickel grid (EMS FF300-Ni). The TEM images were recorded on a JEOL JEM 2100FX TEM instrument (Jeol, Tokyo, Japan) equipped with a GATAN CCD Orius 200D camera (Gatan, Pleasanton, CA, USA) at an acceleration voltage of 200 KV.

### 2.8. Co-Aggregation Test

The co-aggregation experiences were carried out as previously described by Kos et al. [39]. Briefly, the pathogenic strains *E. coli* ATCC 8739, *S. aureus* 2S6 *and L. monocytogenes* 162 as well as *Lactobacillus* strains were cultured at 37°C for 18 h in BHI and MRS broth, respectively. After centrifugation (8000× *g*, 10 min, 4 °C), the pelleted cells were washed twice with sterile PBS (10 mM, pH 7.2), and the cells were re-suspended in PBS to a final concentration of about 10^8^ CFU/mL. Samples of 2 mL of *Lactobacillus* and pathogen suspensions were mixed by vortexing for 30 s in glass test tubes. Tubes containing 4 mL each pathogen suspension or *Lactobacillus* strain alone were considered as controls. Absorbance (A) was measured immediately and after 2 h of incubation at 37 °C. The following formula was used to calculate the co-aggregation percentage:

Co-aggregation (%) = [(Ax + Ay)/2 − A (x + y) ]/[(Ax + Ay)/2] × 100(1)

where A represents the absorbance, x and y represent each strain in the control tubes, and (x + y) represents their mixture.

### 2.9. Inhibition of Pathogenic Strains Adhesion to Caco-2 Cells by Lactobacillus Strains

The human colorectal adenocarcinoma Caco-2 cells were used for the adhesion inhibition assays [40]. The cells were grown at 37 °C in presence of 5% CO_2_ in Dulbeco’s modified Eagle medium (DMEM) containing 4.5 g/L of glucose and supplemented with L-glutamine (2 mM), penicillin (100 U/mL), streptomycin (100 µg/mL), 10% of heat-inactivated fetal bovine serum (FBS) and 1% (*v*/*v*) non-essential amino acids. All these reagents were provided by PAN-Biotech GmbH (Aidenbach, Germany). The adhesion inhibition assays were carried out as described by Bendali et al. [41] including some adjustments. The 24-well tissue culture plates were used to prepare monolayers of Caco-2 cells. The wells were inoculated by 4.10^4^ Caco-2 cells per well, and the plates were incubated for 7 days. Two different protocols were used in order to discriminate competition/exclusion of *E. coli* ATCC 8739, *S. aureus* 2S6 and *L. monocytogenes* 162 with/by lactobacilli.

For exclusion tests, *Lactobacillus* strains at 10^8^ CFU/mL, washed with 1 mL of PBS and resuspended in DMEM without serum or antibiotics, were added to Caco-2 cell monolayers and incubated for 90 min at 37 °C (5% CO_2_). Afterwards, non-adherent *Lactobacillus* strains were removed by washing twice with PBS, and *E. coli* ATCC 8739, *S. aureus* 2S6 or *L. monocytogenes* 162 at 10^7^ CFU/mL, prepared in the same conditions as *Lactobacillus* strains, were added and incubated for an additional 2 h at 37 °C. Here, bacterial charges were added to Caco-2 cells monolayer taking into account the multiplicity of infection (MOI) ratio, in agreement with a previously report [42]. Of note, the MOI ratio of lactobacilli was 1:100 (Caco2: lactobacilli), while that for pathogens was 1:10 (Caco2: pathogens).

For competition tests, *Lactobacillus* (10^8^ CFU/mL) and pathogen (10^7^ CFU/mL) strains, both prepared as previously described, were mixed and added to the Caco-2 monolayers and incubated for 2 h at 37 °C. Then, the Caco-2 monolayers were washed twice with 500 μL of PBS and incubated with 200 μL of Trypsin/EDTA (Gibco) for 15 min to remove Caco-2 cells with adherent bacteria.

After exclusion and competition tests, the enumeration of adherent *E. coli* ATCC 8739, *S. aureus* 2S6 and *L. monocytogenes* 162 cells was performed on specific media for each strain; eosine methylene blue (EMB), Chapman Stone agar and Palcam agar, respectively. Pathogen adhesion rates were calculated in reference to the control (well containing pathogens without *Lactobacillus* strains) which represents 100% adhesion.

### 2.10. CFS, Intact Cells and Intracellular Cell-Free Extract Scavenging Activity on Free Radical DPPH

*Lactobacillus* strains cultures were inoculated into MRS broth and incubated at 30 °C for 18 h and the overnight culture was centrifuged (8000× *g* at 4 °C for 10 min). The cell-free supernatants were subjected to 2,2-di-phényl-2-picrylhydrazyle (DPPH) free radical scavenging assay, as described by Sharma et al. [43]. A volume of 500 µL of cell-free supernatants was added to 3 mL of freshly prepared DPPH (5 mg/100 mL of methanol), mixed by vortexing and incubated for 30 min in the dark. After 30 min, absorbance was measured at 515 nm with non-inoculated MRS broth as blank. Ascorbic acid was used as the synthetic and natural standard. The percentage of radical scavenging activity was calculated according to the equation:

Scavenging (%) = [1 − A515 (sample)/A515 (blank)] 100%(2)

Cells of lactobacilli were harvested by centrifugation (8000× *g* at 4 °C for 10 min) after 18 h of incubation at 30 °C. For the preparation of intact cells, cells were washed three times with PBS (10 mM, pH 7.2) and resuspended at 10^9^ log CFU/mL in this same buffer. To prepare intracellular cell-free extracts, cell pellets were quickly washed twice with deionized water and resuspended in the same solution before transferring to NucleoSpin^®^ Bead Tubes Type B (Macherey-Nagel, Duren, Germany). Tubes were homogenized using FastPrep-24 5G (MP Biomedicals, Santa Ana, CA, USA) for 3 cycles of 30 s, with cooling on ice bath for 5 min between each cycle. Cell debris was removed by centrifugation at 11,000× *g* at 4 °C for 10 min, allowing the recovery of a supernatant containing intracellular cell-free extract [44,45]. As previously described by Lin and Chang, with slight modification, 800 µL of a milliliter of intact cells or intracellular cell-free extract were mixed with 1 mL of freshly prepared DPPH solution (0.004%, *w*/*v* in methanol) and the scavenging ability was calculated as stated above using PBS as a blank.

### 2.11. Statistical Analysis

Differences between samples were calculated using one way ANOVA and the post hoc Tukey test (*p* < 0.05) XL-STAT (version 2009, Addinsoft, Paris, France), and data were expressed as a mean ± standard error calculated from at least three independent experiments. The resulting data were analyzed by principal component analysis (PCA) using FactoMineR software. The principal component analysis was performed using version R 3.5.2 (www.r-project.org, R foundation for statistical computing).

## 3. Results

### 3.1. Lactobacillus Strains Displayed Antibacterial Properties through Different Mechanisms

The ten *Lactobacillus* strains inhibited noteworthy results regarding the growth of the three pathogenic strains used here, according to the results from the spot-on-lawn method (Table 1). Nevertheless, this antagonism was attributed to the non-neutralized CFS based on the well diffusion method. Furthermore, the diameters of the inhibition zones obtained for all *Lactobacillus* strains were similar, regardless of the considered pathogen, except for *Lb. paracasei* FB1 strain, which exhibited lower inhibition zone diameters (Table 1).

### 3.2. Lactic Acid Quantification

The absence of inhibition when the neutralized CFS was used indicated that antibacterial activity was due to the pH lowering, most likely due to the production of lactic acid. Related to that, we noticed that *Lb. plantarum* F3 and 2F8 strains produced the greatest amounts of lactic acid, reaching 16.74 g/L after 24 h of growth. However, the lowest production was registered for *Lb. paracasei* FB1 and *Lb. plantarum* FB3 with 10.73 and 9.64 g/L, respectively (Table 2).

### 3.3. Adhesive Capacity of Lactobacillus and Pathogenic Strains on Polystyrene Microplates

**Figure 1:**
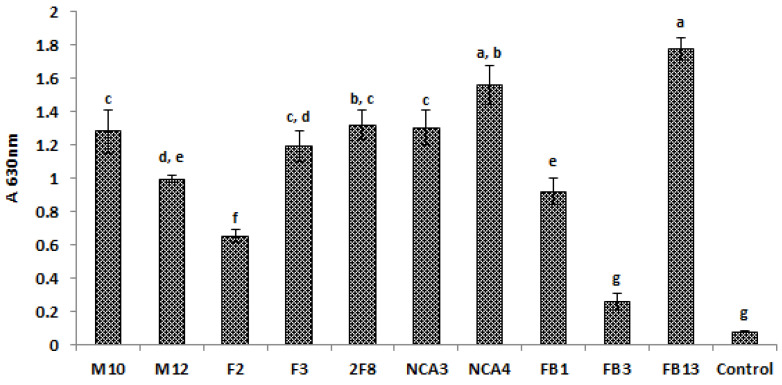
Adhesion of *Lactobacillus* strains to polystyrene plates as determined by A_630nm_ measurements. The absorbance values are the means of three independent experiments. Sterile tryptic soy broth (TSB) was used as a control. The error bars represent the standard deviations. Columns without common letter are significantly different (*p* < 0.05) using Tukey’s test.

The results illustrated in Figure 1 highlight the capabilities of *Lactobacillus* strains to adhere and form biofilms under the tested conditions. Consequently, the absorbencies recorded for *Lactobacillus* strains ranged from 0.26 to 1.78 (Figure 1). Nine out 10 strains were strongly adherent according to the classification proposed by Stepanović et al. [36], whereas *Lb. plantarum* FB3 appeared to be moderately adherent. Remarkably, the most adherent strains were *Lb. plantarum* FB13 followed by *Lb. plantarum* NCA4. Regarding the pathogenic strains, the absorbencies recorded for *E. coli* ATCC 8739, *L. monocytogenes* 162 and *S. aureus* 2S6 were 0.366, 0.422 and 1.368, respectively. Therefore, *S. aureus* 2S6 was thereof considered as strongly adherent, while *E. coli* ATCC 8739 and *L. monocytogenes* 162 were moderately adherent.

### 3.4. The CFS from Lactobacillus Strains Were Able to Prevent the Pathogens Biofilm Formation

**Figure 2:**
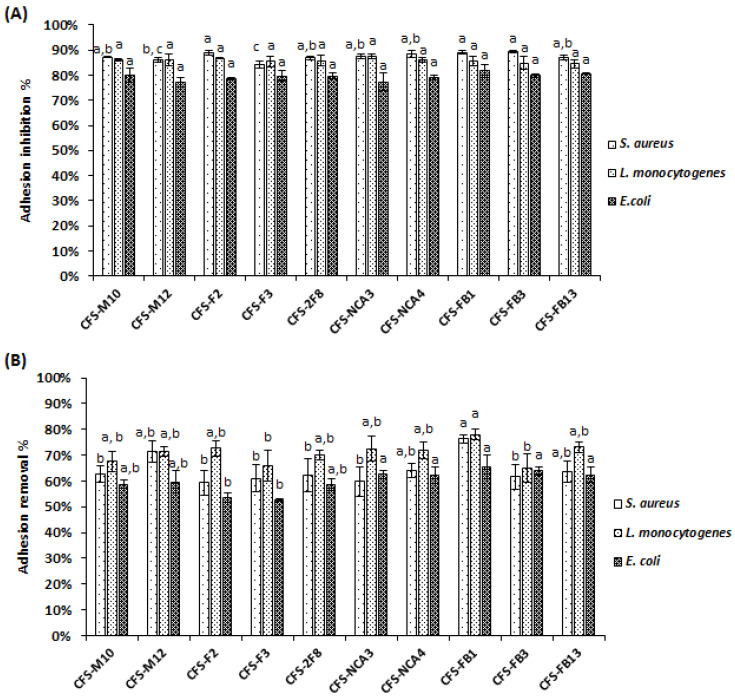
Adhesion inhibition (**A**) and removal (**B**) rates of pathogens (*S. aureus* 2S6, *E. coli* ATCC 8739 and *L. monocytogenes* 162) on polystyrene microplates, by *Lactobacillus* non-neutralized cell-free supernatants. The positive control was the non-treated pathogens suspensions, and the negative control was the pathogens suspensions treated with sterile de Man Rogosa and Sharpe (MRS) broth. The rates are the means of three independent experiments. The error bars represent the standard deviations. Columns without common letter are significantly different (*p* < 0.05) using Tukey’s test.

The non-neutralized CFS from all tested *Lactobacillus* strains were able to prevent the adhesion and subsequently the biofilm formation of *E. coli* ATCC 8739, *S. aureus* 2S6 and *L. monocytogenes* 162 based on the data obtained with the semi quantitative TCP method (Figure 2A). The adhesion inhibition rates ranged between 77.23% ± 1.02% and 89.40% ± 0.41%. The significantly most important percentage of inhibition was registered for *Lb. plantarum* FB3 (89.40% ± 0.41%) against *S. aureus* 2S6 biofilm formation. In addition, these *Lactobacillus* strains were able to remove the formed biofilms after 4 h of contact with the non-neutralized CFS (Figure 2B). Therefore, the biofilm removal levels ranged from 52.60% ± 0.42% to 78.02% ± 1.91%, and the significantly highest rate of removal was shown for *Lb. paracasei* FB1 against *L. monocytogenes* 162 biofilm.

### 3.5. Exopolysaccharide Production

Lactobacilli strains were found to be able to produce 315.55 to 483.22 mg/L of EPS (Table 2). Furthermore, production of EPS in significantly (*p* < 0.05) higher amounts was displayed by *Lb. plantarum* NCA4 (483.22 mg/L), followed by *Lb. plantarum* F2 (454.12 mg/L) and F3 (453.32 mg/L). On the other hand, *Lb. paracasei* FB1 was found to be the lowest EPS producer, with only 315.55 mg/L.

### 3.6. Cellular Ultra-Structural Alterations of Pathogens Treated with CFS Examined by Transmission Electron Microscopy (TEM)

**Figure 3:**
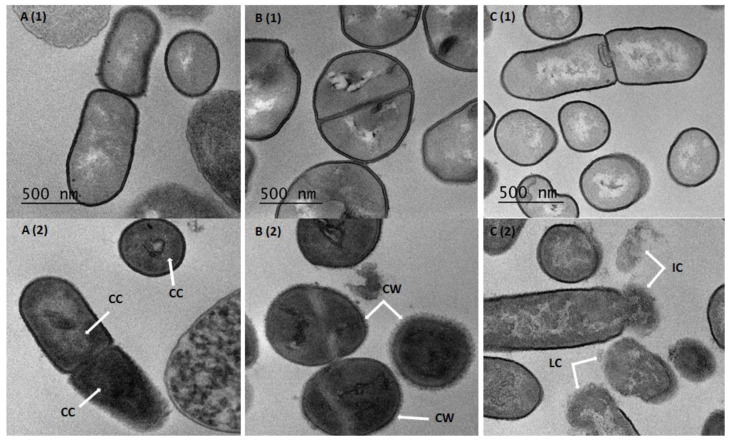
Transmission electron micrographs of non-treated *E. coli* ATCC 8739 (**A**(1)), *S. aureus* 2S6 (**B**(1)) and *L. monocytogenes* 162 (**C**(1)) cells, and treated cells with non-neutralized CFS of *Lb. plantarum* 2F8: *E. coli* ATCC 8739 (**A**(2)), *S. aureus* 2S6 (**B**(2)) and *L. monocytogenes* 162 (**C**(2)). The arrows indicate the main alterations. Abbreviations: cytoplasm contents (CC), cell wall (CW), intracellular contents (IC) and lysed cells (LC).

TEM was used to observe the ultra-structural modifications, if any, of pathogens cells treated with CFS from *Lactobacillus* strains. The data gathered showed the disruption of the cell wall structure and the condensation of ribosomes. Therefore, bacterial death resulted from the wall’s mechanical strength and osmotic lysis. The cytoplasm content of treated *E. coli* cells was agglutinated and appeared as globules of variable size. Nevertheless, the membrane was easily observed as being devoid of any break or clear cut (Figure 3A(2)). Notably, the treated *S. aureus* cells did not show any particular intracellular damage. The cell wall was altered with a “frayed” appearance, indicating a possible peptidoglycan alteration (Figure 3B(2)). Regarding *L. monocytogenes*, cells exhibited an altered cell wall, separation of membrane layers and leakage of intracellular contents (Figure 3C(2)).

### 3.7. The Co-Aggregation Ability of Lactobacilli with Pathogens

**Figure 4:**
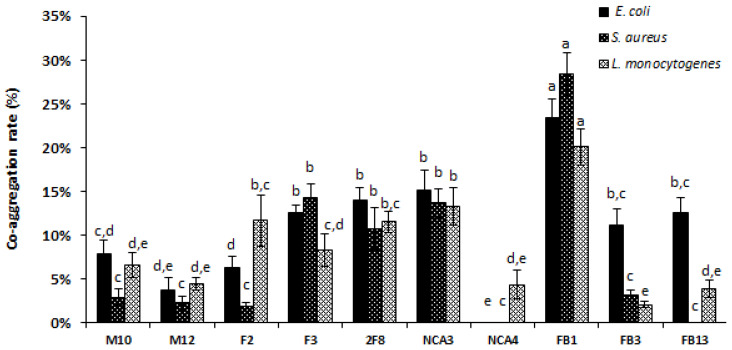
Co-aggregation ability of *Lactobacillus* strains with pathogens after 2 h of process. The rates are the means of three independent experiments. The error bars represent the standard deviations. Columns without a common letter are significantly different (*p* < 0.05) using Tukey’s test.

The co-aggregation of the ten-studied *Lactobacillus* strains with the three pathogens (*E. coli* ATCC 8739, *S. aureus* 2S6 and *L. monocytogenes* 162) is shown in Figure 4. Notably, the co-aggregation registered for *Lb. paracasei* FB1 after 2 h of incubation was significantly (*p* < 0.05) stronger with *S. aureus* (28.34% ± 2.56%), followed by those obtained for *E. coli* (23.39% ± 2.12%), and *L. monocytogenes* (20.10% ± 2.04%). Interestingly, *Lb. plantarum* NCA4 showed co-aggregation with *L. monocytogenes* but not with *E. coli* ATCC 8739 and *S. aureus* 2S6. Similarly, *Lb. plantarum* FB13 did not show co-aggregation with *S. aureus* 2S6.

### 3.8. Anti-Adhesive Activity of Lactobacillus Strains towards Pathogens on Caco-2 Cells

**Figure 5:**
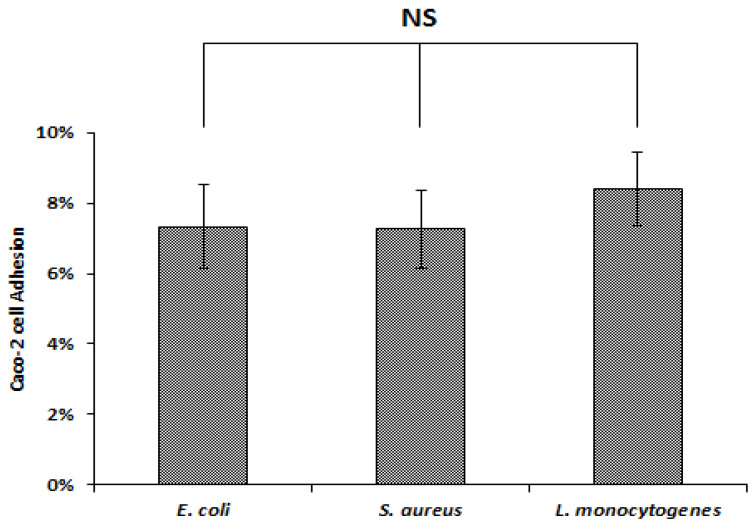
Adhesion percentage of the tested pathogens on Caco-2 cells. Each assay was conducted in triplicate. Means and standard errors are shown. NS, not significantly different (*p* > 0.05) using Tukey’s test.

**Figure 6:**
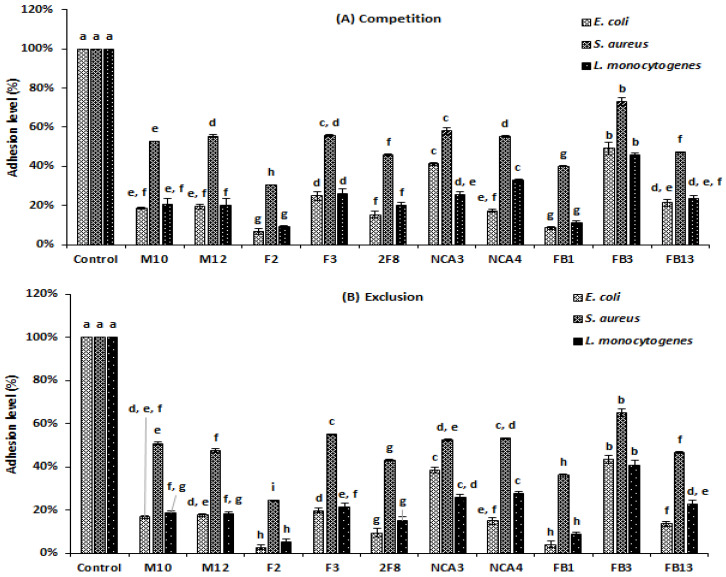
Adhesion rates of *E. coli* ATCC 8739, *S. aureus* 2S6 and *L. monocytogenes* 162 to Caco-2 cells in the presence of *Lactobacillus* strains. A: Competition (*Lactobacillus* strain with pathogen for 120 min). B: Exclusion (pre-incubation of *Lactobacillus* strain for 90 min, before adding pathogen for 120 min). The rates are the means of three independent experiments. The columns without a common letter are different significantly (*p* < 0.05) using Tukey’s test.

The adhesion rates to human epithelial Caco-2 cells of *L. monocytogenes* 162, *S. aureus* 2S6 and *E. coli* ATCC 8739 were 8.40%, 7.25% and 7%, respectively (Figure 5). However, when the adhesion experiment was performed, after incubating either pathogens with *Lactobacillus* strains, the adhesion levels decreased significantly (*p* < 0.05), as depicted in Figure 6. Regarding the competition method, when *Lactobacillus* strains were incubated simultaneously with pathogens, the adhesion rates decreased significantly (*p* ˂ 0.05), ranging from 6.84% to 49.09% for *E. coli* ATCC 8739, 9.05% to 45.74% for *L. monocytogenes* 162, and 30.46% to 72.92% for *S. aureus* 2S6 (Figure 6A). Based on the exclusion method, the adhesion appeared to further decrease. Indeed, when *Lactobacillus* strains were added to Caco-2 cells for 90 min before pathogen incorporation, we observed that rates were between 2.61% and 43.55% for *E. coli* ATCC 8739, 5.13% and 40.89% for *L. monocytogenes* 162 and 24.32% and 65% for *S. aureus* 2S6, as illustrated in Figure 6B. The lowest adhesion rate was reported following addition of *Lb. plantarum* F2 strain, with 2.61%, 5.13%, and 24.32% for *E. coli* ATCC 8739, *L. monocytogenes* 162 and *S. aureus* 2S6, respectively. The presence of *Lb. paracasei* FB1 exhibited a weak adhesion score, with only 3.97% in the presence of *E. coli* ATCC 8739, and this score reached 8.63% in the presence of *L. monocytogenes* 162 and 36.05% for *S. aureus* 2S6.

### 3.9. DPPH Free Radical Scavenging Activity

**Figure 7:**
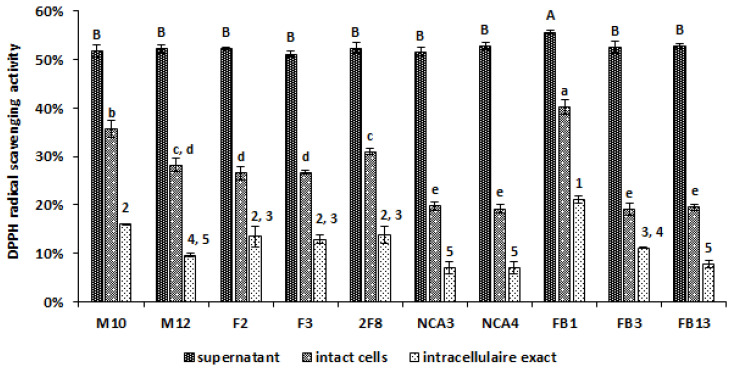
DPPH free radical scavenging activities by supernatants, intact cells and intracellular extract of *Lactobacillus* strains. Each rate was expressed as mean and standard errors are shown. Means within a column with different (number, lowercase and uppercase) letters are significantly different (*p* < 0.05) using one-way ANOVA with Tukey test for pairwise comparisons.

In order to study the antioxidant potential of lactobacilli stains, the DPPH radical scavenging assay was used. Remarkably, the culture supernatants of ten *Lactobacillus* strains exhibited high DPPH scavenging activities, varying from 51.15% ± 0.69% to 55.67% ± 0.77% (Figure 7). We also studied the scavenging activities of the lactobacilli intact cells and intracellular extracts, as shown in Figure 7. The DPPH scavenging rates of intracellular extracts varied between 7.08% ± 1.20% and 21.16% ± 0.69%, and were lower of those of intact cells (19.21% ± 1.28% and 40.18% ± 1.49%). Interestingly, *Lb. paracasei* FB1 strain exhibited the significantly (*p* < 0.05) higher scavenging DPPH rates either for supernatant, intact cells and intracellular extract with 55.67% ± 0.38%, 40.18% ± 1.49% and 21.16% ± 0.69%, respectively, followed by *Lb. plantarum* FB13 supernatant (52.73% ± 0.46%) and *Lb. plantarum* M10 intact cells (35.71% ± 1.68%) and intracellular extract (16.01% ± 0.13%).

### 3.10. Principal Component Analysis and Summary Hierarchical Classification of the Ten Studied Lactobacillus Strains

**Figure 8:**
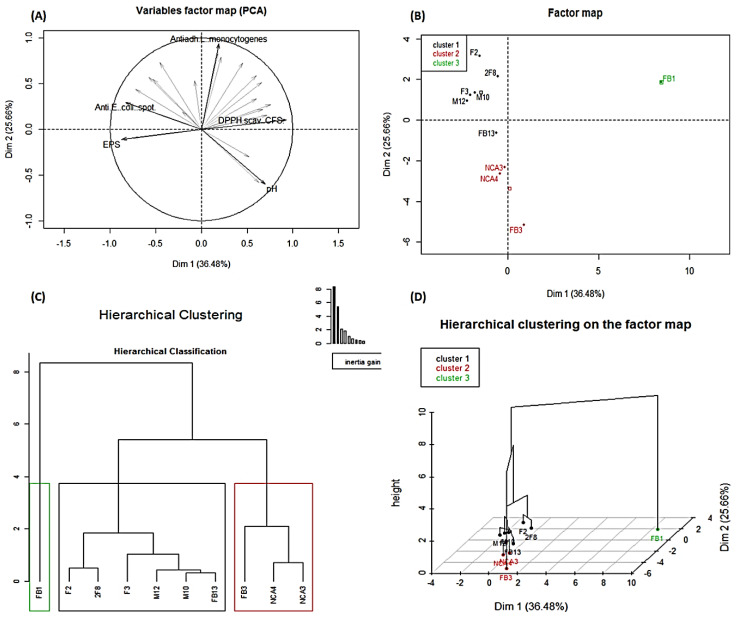
Principal component analysis (PCA) of *Lactobacillus* strains based on the antagonistic, antioxidant and production of both lactic acid and exopolysaccharides using FactoMineR. (**A**) Projection of the 24 variables into the two-dimensional space of Dim 1 and Dim 2; in bold are the variables that contributed most to the construction of the principal component analysis. (**B**) Projection of the ten *Lactobacillus* strains into the two-dimensional space, Dim 1 and Dim 2. (**C**) Hierarchical clustering of the *Lactobacillus* strains generated based on several tested proprieties. (**D**) *Lactobacillus* strains hierarchical grouping in a three-dimensional space.

The results of the PCA performed on the ten lactobacilli strains using 24 variables show that 81.14% of the total variation was distributed in four dimensions (Table S1), and the two most representative dimensions were Dim 1 (36.48%) and Dim 2 (25.66%) (Figure 8A). Thus, it was found that the five variables that most contributed to the construction of this PCA, and consequently to the projection of individuals (lactobacilli) in Figure 8B, were anti-*E. coli* activity (spot method), an antiadhesive effect against *L. monocytogenes* on Caco-2 cells, DPPH scavenging activities of CFS, acidification (pH) and EPS quantification (Figure 8A). Hierarchical principal component classification was performed based on the generated data in this study. Consequently, the lactobacilli were grouped into three main clusters (Figure 8C). The first cluster is composed of six strains, which are *Lb. plantarum* F2, 2F8, M10, M12, F3 and FB13. The second is represented by three strains that are *Lb. plantarum* NCA3, NCA4 and FB3. The variables (Table S2) allowing the classification of these nine strains into two groups are the acidification (pH) and the antibacterial activity against *S. aureus* using the well diffusion method. Indeed, the strains in the first group are significantly the most acidifying (*p* < 0.05) with the greatest antibacterial activity (*p* < 0.05 against *E. coli*, *S. aureus* and *L. monocytogenes* (well-diffusion method) and against *E. coli* (spot method). Meanwhile, the second group of these strains is less acidifying and exhibited a weaker antagonistic activity compared to *Lb. plantarum* strains contained in the first group. However, the third cluster is represented by one strain, namely *Lb. paracasei* FB1, which is significantly (*p* < 0.05) less acidifying, and with lower EPS production and antibacterial activities against *E. coli* and *S. aureus* (using spot method) compared to the other two clusters. This third cluster was significantly (*p* ˂ 0.05) related to (i) high DPPH scavenging activities with CFS and intracellular extract, (ii) co-aggregation with *E. coli*, *S. aureus* and *L. monocytogenes*, and (iii) removing *S. aureus* and *L. monocytogenes* biofilms. Remarkably, the strains in the first and the third cluster had the most significant (*p* ˂ 0.05) antiadhesive activities against *E. coli* and *L. monocytogenes* on Caco-2 cells.

## 4. Discussion

The antagonism and antioxidant effects of *Lactobacillus* strains are key elements in the selection of new strains predicted to be used as bio-preservative agents or qualified as probiotic candidates. Increasing studies have tended to use principal component analysis to select the promising strains [46,47], allowing researchers to group strains with similar characteristics within the same cluster.

The inhibitory properties allocated to *Lactobacillus* strains are due to the production of different antimicrobial substances, such as organic acids that are usually used as food preservatives. Of note, acetic, citric and lactic acids were steadily reported and considered as the most studied organic acids. All of these organic acids are capable of controlling pathogens and stopping their proliferation. They can hamper their capability to form biofilms, or they can act on their quorum signaling pathways [48]. Here, ten novel *Lactobacillus* strains isolated from traditional Algerian fruits were assessed for their inhibitory properties against Gram-positive and Gram-negative pathogenic bacteria, which are representative of main foodborne pathogens. Consequently, the inhibitory properties of these *Lactobacillus* strains were shown to be associated mainly with the production of lactic acid, and exerted in a strain-dependent manner. Of note, these *Lactobacillus* strains of vegetal sources could produce up to 15 g/L of lactic acid, except for *Lb. plantarum* FB3 and *Lb. paracasei* FB1 strains, which produced less than 10.75 g/L. These data fit with those usually reported in the literature. Production of organic acids has several advantages, and was shown, in different studies, to prevent contamination of food during the fermentation process [49,50,51]. Regarding their mode of action, organic acids decrease the intracellular pH of the targeted microorganisms, causing the inhibition of cell growth. More precisely, the addition of organic acids causes proton accumulation in the cytoplasm, which exceeds the buffering levels of the cells and their ability to pump out protons through the H^+^-ATPase transport system [13].

Moreover, all *Lactobacillus* strains considered in this study were able to produce large quantities of EPS, compared to other typical lactobacilli isolated from diverse sources [52,53]. The EPS structure can act as a capsule bound to the cell surface, allowing protection against toxic agents and stress conditions found during desiccation, or osmotic stress and acidity conditions [54,55].

To gain insight on these strains, we established here the role of non-neutralized CFSs obtained from *Lb. plantarum* and *Lb. paracasei* cultures in preventing the initial attachment of pathogenic bacteria such as *E. coli* ATCC 8739, *S. aureus* 2S6 and *L. monocytogenes* 162 to a polystyrene surface. The addition of the non-neutralized CFS has a lasting effect on the biofilm formation by these foodborne pathogens. In direct line, Hossain et al. [56] reported that *Lb. plantarum*, *Lb. curvatus* and *Lb. sakei* isolated from kimchi impeded the biofilm formation by a clinical *L. monocytogenes* strain. Similarly, Mahdi et al. [43] showed the potential of a *Lb. plantarum* strain to prevent the biofilm formation by *E. coli* strains [52]. On the other hand, Cotar et al. [57] reported the role of organic acids, mainly that of lactic acid, produced by *Lb. paracasei* subsp. *paracasei* strain, in the inhibition of the biofilm formation by *Pseudomonas aeruginosa*. All these examples delineate collectively the attributes of *Lactobacillus* strains in controlling the biofilm formation by pathogenic bacteria, through the production of organic acids. Nevertheless, these studies failed in showing the anti-adhesive properties of the *Lactobacillus* strains used.

Furthermore, we examined here the morphology and the cell structure of pathogenic bacteria following their treatments with the non-neutralized CFS using TEM. We then noticed clear changes in the global morphology, even in the cell ultra-structure of the aforementioned target bacteria, when they were treated with the CFS *Lb. plantarum* 2F8 strain that was characterized as one of the upmost strains for lactic acid production. Moreover, *S. aureus* 2S6 and *E. coli* ATCC 8739 were morphologically altered, whereas *L. monocytogenes* 162 exhibited a disruption of its cell membrane integrity. Similar alterations were previously observed for human pathogenic bacteria including staphylococci, enterotoxigenic enterobacteria, *Candida albicans*, *Acinetobacter baumannii* and *L. monocytogenes* treated with different *Lactobacillus* species like *Limosilactobacillus fermentum*, *Lactobacillus jensenii*, *Lacticaseibacillus rhamnosus* and *Lacticaseibacillus paracasei* species [58,59,60].

The *Lactobacillus* strains used here prevented the adhesion of *E. coli* ATCC 8739, *S. aureus* 2S6 and *L. monocytogenes* 162 on eukaryotic Caco-2 cells, when used at an appropriate MOI ratio. In direct line with this, Yu et al. [61] reported the preventive effect of lactobacilli against enteric pathogenic bacteria using the same ratio of 10:1 (lactobacilli/pathogens). The anti-adhesive property of these *Lactobacillus* strains was dependent on many factors, including the conditions of incubation of Lactobacilli and pathogens during the competition and exclusion assays. Of note, the invasion of eukaryotic Caco-2 cells by *L. monocytogenes*, which is an intracellular pathogen, can be as well affected by these adherent *Lactobacillus* strains. The data gathered from the exclusion test are promising since they pointed out a clear protective effect against these pathogens, mainly by impeding their adhesion on the Caco-2 monolayer model, which strengthens our former report [34]. Of note, the abilities to hamper pathogens adhesion to the host tissues is as a key hurdle, and can therefore prevent their invasion process or limit some of their adverse effects, such as the destabilization of intercellular junctions [62].

In agreement with probiotic selection, *Lactobacillus* strains are known to prevent the adhesion of pathogenic bacteria to biotic surfaces by employing different mechanisms, other than the production of bioactive metabolites [63]. Among these mechanisms, the co-aggregation considered as a key strategy [39,64] is of major interest. Here, the co-aggregation rate varied widely from one *Lactobacillus* strain to another. Thus *Lb. plantarum* NCA4 and FB13 strains lack this function, while *Lb. paracasei* FB1 displayed a co-aggregation rate of over 20% with all target bacteria tested. In good agreement with this, *Lactobacillus* strains were shown to control through the co-aggregation process the microenvironment around the pathogenic cells [64]. Beganović et al. [65], Ferrando et al. [66] reported that surface proteins of *Lactobacillus* strains were involved in both auto-aggregation and co-aggregation with pathogens. Thus, co-aggregation, as above-indicated, is a strategic function which can be involved in competitive and exclusion mechanisms, leading thereof to a reduction in pathogenic load during infections [67].

Among the interesting probiotics aspects, some *Lactobacillus* strains have been reported to have antioxidant properties by decreasing the level of oxidants such as reactive oxygen species (ROS) [68]. The DPPH assay is generally used in vitro to determine the scavenging activity, and is one of the most sensitive, common and reliable methods [69]. In this study, the lactobacilli antioxidant capacity was focused on intact cells, cell-free supernatants and intracellular extracts. The important DPPH scavenging rates using cell-free supernatants are consistent with those reported by Sharma et al. [43]. Otherwise, the DPPH scavenging activity of intact cells was greater than the rates recorded for the intracellular extracts, as reported in previous studies [70,71]. The intracellular extract can have a significant antioxidant postbiotic effect [72,73]. Many studies have revealed that antioxidant activity of *Lactobacillus* strains might be linked to their production of cell-surface compounds, e.g., lipoteichoic acid and EPS, and to antioxidant enzymes, such as superoxide dismutase, NADH-oxidase and NADH-peroxide, and heterologous non-haem catalase [74,75].

## 5. Conclusions

Here, we characterized the antibacterial, anti-adhesive and antioxidant properties of ten *Lactobacillus* strains from vegetal sources, and confirmed their potential to produce lactic acid and EPS in a strain-dependent manner. In addition, we revealed, using TEM, accurate ultra-structure damages caused by lactic acid produced by these strains. These *Lactobacillus* strains exhibited capabilities to adhere on human Caco-2 cells, as well as on inanimate surfaces; this mechanism stands as a hurdle to stop the proliferation of pathogens such as *E. coli*, *S. aureus* and *L. monocytogenes*. Collectively, the results from this study open up new possibilities for application of these new strains as potential probiotics or bioprotective cultures. For the probiotic application, they can be used, after further assessments, to protect human or animal GIT microbiota from malevolent pathogens and oxidative stress. For their application as bioprotective cultures, they can indeed be employed as a hurdle mean, mainly in processed foods from vegetal origin to replace chemicals and ascertain their safety. For both applications, further in vivo and in situ evidence is needed, and this will be our next focus.

## Figures and Tables

**Table 1:** Inhibition of pathogenic bacteria by *Lactobacillus* strains based on two different methods.

Strain	Diameter of Inhibition Zones Given in Millimeters (mm)						Supernatants pH
	Spot-on-lawn Method			Wells Diffusion Method			
	*E. coli* ATCC 8739	*S. aureus* 2S6	*L. monocytogenes* 162	*E. coli* ATCC 8739	*S. aureus* 2S6	*L. monocytogenes* 162	
M10	43± 1.00 ^a^	31 ± 1.04 ^a,b^	21 ± 1.04 ^d^	14 ± 0.29 ^a^	13 ± 1.00 ^a^	13 ± 0.35 ^a^	3.81 ± 0.00 ^c,d^
M12	41 ± 1.52 ^a^	31 ± 1.00 ^a,b^	23 ± 1.04 ^b,c,d^	13 ± 0.35 ^a,b^	13 ± 0.50 ^a^	13 ± 0.16 ^a^	3.79 ± 0.02 ^c,d^
F2	41 ± 1.51 ^a,b^	30 ± 1.23 ^a,b^	25 ± 0.50 ^a,b^	14 ± 0.50 ^a^	13 ± 0.46 ^a^	12 ± 0.17 ^a,b^	3.83 ± 0.01 ^c^
F3	34 ± 0.51 ^c,d^	28 ± 1.14 ^b^	24 ± 1.04 ^a,b,c^	14 ± 0.60 ^a^	13 ± 0.29 ^a^	12 ± 0.29 ^a,b^	3.77 ± 0.02 ^d^
2F8	42 ± 1.72 ^a^	31 ± 1.30 ^a,b^	27 ± 0.50 ^a^	14 ± 0.80 ^a^	13 ± 0.23 ^a^	12 ± 0.35^a,b^	3.79 ± 0.02 ^c,d^
NCA3	37 ± 1.50 ^b,c^	30 ± 1.00 ^a,b^	23 ± 1.52 ^b,c,d^	12 ± 0.60 ^b^	10 ± 0.57 ^c^	11 ± 0.29 ^b^	3.94 ± 0.01 ^b^
NCA4	36 ± 1.00 ^c,d^	29 ± 1.23 ^a,b^	22 ± 1.15 ^b,c,d^	12 ± 1.00 ^b^	10 ± 0.57 ^c^	11 ± 0.29 ^b^	3.98 ± 0.02 ^b^
FB1	25 ± 2.00 ^e^	23 ± 0.20 ^c^	23 ± 0.50 ^b,c,d^	12 ± 0.30 ^b^	11 ± 0.46 ^b,c^	11 ± 0.17 ^b^	4.07 ± 0.01 ^a^
FB3	32 ± 1.60 ^d^	31 ± 0.91 ^a^	22 ± 0.50 ^c,d^	12 ± 1.00 ^b^	11 ± 0.29 ^b,c^	11 ± 0.50 ^b^	4.10 ± 0.00 ^a^
FB13	37 ± 1.80 ^b,c^	31 ± 1.00 ^a,b^	22 ± 1.50 ^c,d^	13 ± 0.65 ^a,b^	12 ± 0.35 ^a,b^	12 ± 1.00 ^a,b^	3.78 ± 0.01 ^d^

Results are expressed as mean ± standard deviation *n* = 3, means with different lowercase letters were significantly different (*p* < 0.05) based on Tukey’s test.

**Table 2:** Lactic acid and exopolysaccharides (EPS) production in *Lactobacillus* strains.

Strain	Lactic Acid Quantification (g/L)			EPS Production (mg/L)
	8 h	18 h	24 h	
M10	4.18 ± 0.06 ^b^	12.4 ± 0.64 ^a,b^	15.28 ± 0.13 ^b^	424.48 ± 23.59 ^b,c^
M12	3.76 ± 0.06 ^c^	13.20 ± 0.28 ^a^	16.15 ± 0.18 ^a,b^	436.47 ± 14.82 ^a,b,c^
F2	4.27 ± 0.06 ^b^	11.05 ± 1.03 ^b,c^	15.88 ± 0.22 ^a,b^	454.12 ± 25.53 ^a,b^
F3	4.86 ± 0.04 ^a^	12.55 ± 0.43 ^a^	16.74 ± 0.36 ^a^	453.32 ± 17.55 ^a,b^
2F8	4.58 ± 0.07 ^a^	12.84 ± 0.14 ^a^	16.74 ± 0.30 ^a^	425.13 ± 11.81 ^b,c^
NCA3	4.17 ± 0.24 ^b^	12.85 ± 0.26 ^a^	15.19 ± 0.24 ^a,b^	433.18 ± 14.57 ^b,c^
NCA4	4.77 ± 0.02 ^a^	13.65 ± 0.13 ^a^	16.21 ± 0.62 ^a,b^	483.22 ± 15.39 ^a^
FB1	3.01 ± 0.09 ^e^	9.88 ± 0.36 ^c^	10.73 ± 0.37 ^c^	315.55 ± 13.87 ^d^
FB3	2.59 ± 0.13 ^f^	7.18 ± 0.36 ^d^	9.64 ± 0.33 ^d^	402.22 ± 18.76 ^c^
FB13	3.40 ± 0.07 ^d^	12.57 ± 0.25 ^a^	15.67 ± 0.35 ^b^	412.69 ± 10.69 ^b,c^

Results are expressed as mean ± standard deviation *n* = 3, means with different lowercase letters were significantly different (*p* < 0.05) based on Tukey’s test.

## Supplementary Materials

The following are available online at https://www.mdpi.com/2304-8158/9/8/985/s1. Table S1: Correlation, contribution and representation of the variables with the dimensions of the summary PCA based on factor loadings. Table S2: Description of each Lactobacillus cluster by quantitative variables.
