# Supplementary material for: Clusters of Lactobacillus Strains from Vegetal Origins Are Associated with Beneficial Functions: Experimental Data and Statistical Interpretations
Source: Foods. 2020 Jul 24;9(8):985. doi: 10.3390/foods9080985 (PMC7466302; doi:10.3390/foods9080985)
Supplement: Supplementary file 1 [file foods-09-00985-s001.pdf]

Supplementary data

Table S1. Correlation, contribution and representation of the variables with the dimensions of the summary PCA based on factor loadings.

Variable	Correlation				Contribution (%)				cos^2			
	Dim1	Dim2	Dim3	Dim4	Dim1	Dim2	Dim3	Dim4	Dim1	Dim2	Dim3	Dim4
Antibfm *E. coli*	0.608	0.121	−0.578	0.090	4.21	0.236	12.47	0.433	0.369	0.015	0.335	0.008
Antibflm *S. aureus*	0.521	−0.302	0.271	0.404	3.09	1.482	2.732	8.658	0.271	0.091	0.073	0.163
Antibfm *L. monocytogens*	−0.207	0.189	0.779	−0.188	0.49	0.583	22.60	1.878	0.043	0.036	0.606	0.035
Rvbfm *E. coli*	0.626	−0.581	0.037	0.277	4.48	5.476	0.051	4.080	0.392	0.337	0.001	0.077
Rvbfm *S. aureus*	0.669	0.220	−0.135	0.352	5.11	0.785	0.676	6.584	0.448	0.048	0.018	0.124
Rvbfm *L. monocytogens*	0.602	0.334	0.515	0.355	4.14	1.810	9.888	6.698	0.363	0.111	0.265	0.126
**Anti- *E. coli* (spot)**	−0.827	0.291	0.139	0.164	7.81	1.373	0.719	1.435	0.685	0.085	0.019	0.027
Anti- *S. aureus* (spot)	−0.748	−0.097	−0.178	−0.020	6.39	0.154	1.179	0.022	0.560	0.009	0.032	0.000
Anti- *L. monocytogens* (spot)	−0.072	0.529	0.301	−0.428	0.06	4.544	3.385	9.698	0.005	0.280	0.091	0.183
Anti- *E. coli* (well)	−0.526	0.548	−0.332	0.050	3.16	4.873	4.111	0.134	0.276	0.300	0.110	0.003
Anti- *S. aureus* (well)	−0.450	0.728	−0.448	−0.009	2.32	8.602	7.485	0.004	0.203	0.530	0.201	0.000
Anti- *L. monocytogens* (well)	−0.576	0.568	−0.379	0.235	3.79	5.246	5.351	2.938	0.331	0.323	0.144	0.055
**pH**	0.701	−0.602	0.121	0.018	5.60	5.877	0.542	0.018	0.491	0.362	0.015	0.000
Antiadh. *E. coli*	0.178	0.828	0.082	0.395	0.36	11.14	0.249	8.282	0.032	0.686	0.007	0.156
Antiadh. *S. aureus*	0.213	0.787	0.387	0.249	0.52	10.05	5.594	3.285	0.045	0.619	0.150	0.062
**Antiadh. *L. monocytogens***	0.194	0.938	0.242	0.114	0.43	14.29	2.177	0.687	0.038	0.880	0.058	0.013
Co-agg. *E. coli*	0.714	0.153	−0.175	−0.523	5.82	0.378	1.141	14.48	0.509	0.023	0.031	0.273
Co-agg. *S. aureus*	0.753	0.272	0.035	−0.556	6.48	1.204	0.045	16.42	0.567	0.074	0.001	0.310
Co-agg. *L. monocytogens*	0.658	0.514	0.372	−0.372	4.94	4.297	5.156	7.349	0.433	0.265	0.138	0.139
Lactic acid production	−0.718	0.435	0.295	−0.006	5.89	3.071	3.252	0.002	0.516	0.189	0.087	0.000
**DPPH scav (CFS)**	0.926	0.105	−0.014	0.337	9.98	0.187	0.008	6.022	0.858	0.011	0.000	0.114
DPPH scav (IC)	0.452	0.725	−0.267	−0.028	2.33	8.532	2.658	0.042	0.204	0.525	0.071	0.001
DPPH scav (ICE)	0.598	0.588	−0.357	−0.121	4.09	5.618	4.743	0.772	0.358	0.346	0.127	0.015
**EPS quantification**	−0.871	−0.109	0.318	0.037	8.67	0.195	3.781	0.074	0.759	0.012	0.101	0.001

Abbreviation: Dimension (Dim), representation quality (cos2), anti-biofilm (Antibfm), remove biofilm (Rvbfm), spot-on-lawn method (spot), well diffusion method (well), antiadhesive on Caco-2 cells (Antiadh), co-aggregation (Co-agg), scavenging activity (scav), cell free supernatant (CFS), intact cells (IC) and intracellular extract (ICE). In bold the variables that contributed most to the construction of the principal component analysis.

Table S2. Description of each *Lactobacillus* cluster by quantitative variables**.**

Cluster	Variable	V Test	Mean Category	Overall Mean	*P* Value
Cluster 1: *Lb. plantarum* F2, 2F8, M10, M12, F3 and FB13	Anti- *S. aureus* (well)	2.80	12.83 mm	11.90mm	0.0049
	Anti- *L. monocytogens* (well)	2.61	12.33 mm	11.80 mm	0.0088
	Anti- *E. coli* (well)	2.24	13.33 mm	12.80 mm	0.024
	Anti- *E. coli* (spot)	2.02	39.66 mm	36.80 mm	0.043
	Rvbfm *E. coli*	−2.15	57.61%	59.96%	0.031
	pH	−2.79	3.79	3.88	0.00542
Cluster 2: *Lb. plantarum* NCA3, NCA4 and FB3	pH	1.97	4.00	3.88	0.047
	DPPH scav (IC)	−2.01	19.39%	26.61%	0.043
	Anti- *L. monocytogens* (well)	−2.09	11 mm	11.80 mm	0.035
	Antiadh. *E. coli*	−2.20	67.68%	81.94%	0.027
	Antiadh. *L. monocytogens*	−2.24	68.65%	79.57%	0.024
	Anti- *S. aureus* (well)	−2.52	10.33 mm	11.90 mm	0.011
Cluster 3: *Lb. paracasei* FB1	DPPH scav (CFS)	2.72	55.67%	52.50%	0.0063
	Co-agg. *S. aureus*	2.39	28.34%	7.71%	0.016
	Rvbfm *S. aureus*	2.33	76.27%	64.25%	0.019
	Co-agg. *L. monocytogens*	2.18	20.10%	8.63%	0.029
	DPPH scav (ICE)	2.16	21.16%	12.02%	0.030
	Co-agg. *E. coli*	2.03	23.38%	10.67%	0.041
	Rvbfm *L. monocytogens*	1.96	78.01%	70.80%	0.049
	Anti- *E. coli* (spot)	−2.26	25 mm	36.80 mm	0.023
	Anti- *S. aureus* (spot)	−2.38	23 mm	29.20 mm	0.017
	EPS	−2.56	315.33 mg/l	424.04	0.010

Abbreviation: V. test (Kuiper's test), remove biofilm (Rvbfm), spot-on-lawn method (spot), well diffusion method (well), antiadhesive on Caco-2 cells (Antiadh), co-aggregation (Co-agg), scavenging activity (scav), cell free supernatant (CFS), intact cells (IC) and intracellular extract (ICE). ($P < 0.05$) were significantly different determined with Principal Component Analysis (PCA) using FactoMineR software.
